# The Influence of Tumor-Specific Markers in Breast Cancer on Other Blood Parameters

**DOI:** 10.3390/life14040458

**Published:** 2024-03-29

**Authors:** Vlad Bogdan Varzaru, Anca-Elena Eftenoiu, Daliborca Cristina Vlad, Cristian Sebastian Vlad, Aurica Elisabeta Moatar, Roxana Popescu, Ionut Marcel Cobec

**Affiliations:** 1 Doctoral School, Faculty of Medicine, “Victor Babes” University of Medicine and Pharmacy Timisoara, 300041 Timisoara, Romania; 2 ANAPATMOL Research Center, Faculty of Medicine, “Victor Babes” University of Medicine and Pharmacy Timisoara, 300041 Timisoara, Romania; 3 Department of Medical Genetics, “Carol Davila” University of Medicine and Pharmacy Bucharest, 050474 Bucharest, Romania; 4 Department of Pharmacology, Faculty of Medicine, “Victor Babes” University of Medicine and Pharmacy Timisoara, 300041 Timisoara, Romania; 5 Emergency County Clinical Hospital Pius Brinzeu Timisoara, 300723 Timisoara, Romania; 6 Clinic of Internal Medicine-Cardiology, Klinikum Freudenstadt, 72250 Freudenstadt, Germany; 7 Clinic of Obstetrics and Gynecology, Klinikum Freudenstadt, 72250 Freudenstadt, Germany

**Keywords:** breast cancer, women, tumor markers, blood test parameters

## Abstract

Background: Breast cancer is the most frequently diagnosed cancer among women, responsible for the highest number of cancer-related deaths worldwide. There is limited data available related to serum tumor markers in breast cancer and other blood parameters or other glandular laboratory parameters. This study aims to evaluate the correlation of tumor-specific markers for breast cancer with other blood parameters and how these correlations could impact clinical management. Material and Method: This retrospective study represents a data analysis from 1 January 2020 to 31 May 2023, in the County Hospital of Timisoara, Romania. We reviewed all the cases where, in the laboratory analyses, the serum tumor specific biomarkers for breast cancer were analyzed. Results: A statistical analysis was performed in order to identify a possible relationship between CA 15-3 and the various biomarkers and blood parameters included in the present study. Values were classified according to reference ranges. The tests revealed no statistically significant associations between CA 15-3 values and the levels of CA125 (χ^2^(1) = 1.852, *p* = 0.174), CEA (χ^2^(1) = 1.139, *p* = 0.286), AFP (Fisher’s exact test, *p* = 0.341), fT4 (Fisher’s exact test, *p* = 0.310), TSH (Fisher’s exact test, *p* = 0.177), or PTH (Fisher’s exact test, *p* = 0.650). Conclusion: The findings indicate a lack of strong correlation between CA 15-3 and CA125, CEA, AFP, thyroid function markers, or PTH within this cohort.

## 1. Introduction

Breast cancer is the most frequently diagnosed cancer among women, being responsible for the highest number of cancer-related deaths worldwide [1,2]. Despite the availability of early detection methods and complex therapy options, it remains a major source of morbidity and mortality globally [3].

Breast imaging plays a key role in detecting and monitoring breast tumors [4,5]. Before obtaining additional imaging, breast cancer may progress with ineffective treatment. In some situations, imaging fails to show progression at an early stage. Biomarkers, especially serum biomarkers, can provide additional insight or even detect disease progression or response to treatment before imaging can [6]. Although the primary diagnostic techniques for early breast cancer include imaging, ultrasound, and pathology, these methods are subject to variability because of subjective factors, for example, the medical practice experience and the current state of technological advancements [7]. Tissue expression biomarkers in breast cancer, such as estrogen receptor (ER), progesterone receptor (PR), and human epidermal growth factor receptor 2 (HER2), are extensively used as tumor-specific markers to guide breast cancer therapy. 

On the other hand, in a practical approach, at the St. Gallen International Breast Cancer Conferences in 2011 and 2013, it was agreed that the different breast cancer intrinsic subtypes can be defined not only by genetic array testing but also by an approximation using immunohistochemistry. A definition of intrinsic subtypes of breast cancer was proposed: luminal A (ER+ and/or PR+, Ki67 low, and Her2−), luminal B (ER+ and/or PR+, Ki67 high, and/or Her2+), Her2-positive (ER-, PR-, and Her2+), and triple-negative (ER-, PR-, Her2-) [8,9].

Current clinical tests require tumor tissue obtained through biopsy or other surgical approaches. Non-invasive biomarkers are desired for early detection, prognosis, detecting progressive disease, and monitoring therapy response in breast cancer. Blood, rich in molecular and cellular elements, holds great promise as a compartment for developing non-invasive cancer diagnostics. Serum biomarkers, in this regard, have the potential to assess the disease and an individual’s health status [10,11,12,13].

The implementation of serum biomarkers, characterized by low cost, easy acquisition, and wide usage in the clinical setting, represents a convenient clinical development. However, the reliance on single biomarkers has limitations, and the optimal combination of biomarkers is still unclear [7]. The most commonly used biomarkers in relation to breast cancer are carcinoembryonic antigen (CEA), alpha fetoprotein (AFP), carbohydrate antigen 125 (CA125), carbohydrate antigen 199 (CA199), and carbohydrate antigen 15-3 (CA15-3) [7].

Currently, the European Society for Medical Oncology (ESMO) Clinical Practice Guidelines do not recommend tumor markers such as CA15-3 or CEA for follow-up in early breast cancer. In the case of advanced breast cancer, according to the guidelines developed by the European School of Oncology in collaboration with ESMO, the utility of tumor markers in diagnosis and adjuvant treatment follow-up is unclear. Nevertheless, they can be helpful in evaluating treatment responses, especially in non-measurable metastatic disease. It is important to note that treatment decisions should not be based exclusively on a change in tumor markers [14,15].

The American Society of Clinical Oncology (ASCO) does not recommend tumor markers such as CA15-3 or CA27.29 for screening, diagnosis, staging, and monitoring for recurrence in breast cancer. The significance of such tumor markers in early breast cancer remains uncertain, although they may have prognostic value. CA15-3 or CA27.29 alone should not be used for treatment monitoring, although increasing levels may indicate unsuccessful treatment in situations where disease progression is not measurable. CEA, CA15-3, and CA27.29 may be used to aid treatment decision-making in metastatic breast cancer, but they should not be used alone for the evaluation of treatment efficacy [16].

Circulating markers in breast cancer serve as a valuable tool for patient prognosis and monitoring therapy response, particularly in disease recurrence, progression, and metastatic disease. According to the literature, serum biomarkers CA15-3, CA27-29, and CEA have low sensitivity in early breast cancer. However, their sensitivity increases, and they become useful as serial measurements in managing metastatic breast cancer and monitoring therapy responses in progressive disease settings [6]. The diagnostic accuracy of single blood biomarker detection is less than ideal, but combining measurements of blood biomarkers could increase the accuracy of diagnosing breast cancer recurrence [7,17,18,19].

CEA, a glycoprotein involved in cell adhesion, can be elevated in various cancers, while CA15-3, a protein antigen of the transmembrane glycoprotein MUC-1, increases in higher breast cancer stages [20,21,22,23]. CEA is naturally occurring during fetal development and may also be present only in trace amounts in healthy adults. CEA, found on the surface of malignant tumor cells, is recognized as a broad-spectrum tumor marker, used in treatment monitoring and prognosis assessment, and is characteristic for colorectal cancer while having a low specificity in other cancers [7,24]. 

Breast cancer, a heterogeneous disease, expresses various aberrant proteins capable of eliciting an immune response that may manifest months or years before clinical diagnosis [25,26]. Another serum biomarker, CA 125, is a glycoprotein whose production has been demonstrated in healthy breast tissue, though its significance in breast cancer elevation remains uncertain. Elevated serum levels of CA 125 are associated with increasing bulk disease and a poor prognosis in the metastatic setting. Additionally, they are linked with epithelial ovarian cancer, other tumors, and non-malignant diseases [27,28,29,30]. Some studies recommend measuring CA 15-3 serum values in conjunction with other tumor markers [31]. 

CA125, or MUC16 protein, is a high molecular weight glycoprotein on the cell surface characteristic of ovarian cancer, fallopian tube cancer, endometrial cancer, cervical cancer, pancreatic cancer, liver cancer, lung cancer, and digestive tumors, according to the literature [7]. 

Data on associations between breast cancer and other glands are scarce and conflicting, especially regarding the associations between thyroid dysfunction and breast cancer risk. Considering the role of thyroid hormones in cell proliferation and differentiation, thyroid dysfunction is considered a potential risk factor for breast cancer [32,33,34,35]. Some studies associate hypothyroidism and hyperthyroidism with a lower and higher risk of breast cancer, respectively, while others link hyperthyroidism to a significantly increased risk of breast cancer mortality [27,36].

α-fetoprotein (AFP) is physiologically synthesized during pregnancy, but is also expressed in various tumors, such as hepatocellular carcinoma, intrahepatic cholangiocarcinoma, gastric cancer, and germ cell tumors (testicular and ovarian) [37]. Its primary use as a tumor marker is in the screening, diagnosis, and follow-up of hepatocellular carcinoma, but it can also exhibit high concentrations in other malignancies [7].

Limited data are available on circadian rhythmicity, serum cortisol, and tumor marker antigens in breast and ovarian cancer [38]. Parathyroid hormone-related protein (PTHrP), found in breast cancer, is produced by mammary epithelial cells. The characteristics of PTHrP as a tumor marker in advanced breast cancer did not match those of the established markers CA15-3, CEA, and ESR [39].

The action of the pituitary hormone and thyroid stimulating hormone (TSH) on the thyroid gland results in the production of thyroid hormones, namely, thyroxine (T4) and triiodothyronine (T3). After being released into the circulation, T4 is transformed into T3 within target cells. Cancer pathogenesis is influenced by the hypothalamic–pituitary–thyroid axis through altering systemic factors [40,41]. Elevated levels of thyroid hormones have been associated with an increased risk of solid tumors, including breast cancer [34,41]. Research indicates that in breast cancer, T4 and T3 levels are typically high, while TSH levels are low [40,42]. Currently, the link between thyroid diseases and breast cancer is still unclear. Some studies report that hyperthyroidism is associated with an increased risk of breast cancer [43,44], while other studies found no significant relationship between hyperthyroidism or hypothyroidism and breast cancer [35,42]. High serum levels of T4 and TSH were associated with hormone receptor-positive and HER2-negative breast cancers [40]. 

There is limited available data regarding serum tumor markers in breast cancer in relation to other blood parameters or glandular laboratory parameters. The purpose of this study is to evaluate the correlation of tumor-specific markers for breast cancer with other blood parameters and how these correlations could impact clinical management.

## 2. Materials and Methods

This retrospective study represents an analysis of breast cancer patient data from the County Hospital of Timisoara, Romania. We analyzed the records of female patients with breast cancer who underwent testing for CA 15-3, CEA, CA125, AFP, TSH, FT4, and PTH from 1 January 2020 to 31 May 2023, in Romania, in the context of breast cancer diagnosis and/or treatment.

The focus was primarily on individuals for whom CA 15-3, a recognized marker for breast cancer, was measured. Thus, we excluded cases where CA15-3 was not measured. Additionally, efforts were made to identify cases in which CEA, CA125, AFP, and thyroid function were assessed. In situations where multiple measurements of a specific marker were available for a single case, only the highest recorded value was included for analysis.

The data collection process involved the use of Microsoft Office Excel 2019 for organizing and storing information. Subsequently, data analysis was conducted using SPSS IBM Statistics 20 software. Statistical analyses were performed using the chi-square test or Fisher’s exact test. A significance threshold of *p* ≤ 0.05 was established to determine statistical significance. The graphical representation was created using Microsoft Office Excel.

Variables were categorized based on the reference laboratory values of the hospital:

- CA 15-3: 0–32.4 U/mL
- CEA: <10 ng/mL
- CA125: 0–30.2 U/mL
- AFP: 0–8.1 ng/mL
- FT4: 11.50–22.70 pmol/L
- TSH: 0.55–4.78 mlU/L
- PTH: 18.5–88 pg/mL

The analysis of all studied parameters was performed on the ADVIA Centaur XPT immunoassay using direct chemiluminescent technology, and the reference interval was previously established and set by the manufacturer, Siemens. All the information about the method, reagents, specimen collection and handling, and reference values is described in detail in the insert of reagent kits and is according to CLSI (Clinical and Laboratory Standards Institute).

The analysis aimed to find significant relationships between CA 15-3 and other circulating markers, with implications for understanding their interplay in the context of breast cancer.

## 3. Results

We performed a descriptive analysis for each biomarker included in the study: CA15-3, CEA, CA125, AFP, and thyroid function. Mean, median, standard deviation (SD), and minimum and maximum values are reported in Table 1.

CA 15-3 displayed a mean value of 111.42, ranging from a minimum of 4.50 to a maximum of 4000.00, showing substantial variability (SD = 317.74). CEA demonstrated a mean value of 24.18 (SD = 100.11), with a range from 0 to 1130.0, whereas CA125 showed a mean of 337.63 (SD = 916.43), varying from 0 to 5530.00. The mean of AFP values was 361.79 (SD = 2918.29), ranging from 0 to 29,800.00.

The thyroid function markers, fT3, fT4, and TSH, had means of 4.15 (SD = 1.32), 18.07 (SD = 5.50), and 3.35 (SD = 3.44), respectively. The mean cortisol level was 29.51 (SD = 17.55), varying from 10.40 to 70.66, while PTH had a mean of 163.47 (SD = 175.34), varying from 7.90 to 672.50.

Table 2 summarizes the absolute and relative frequencies for the studied variables. Variables were grouped into different categories (positive/negative) or levels (low, normal, and high) based on their registered value.

In the studied sample, 83.5% had CA 15-3 values greater than 32.4 U/mL. CEA values were below 10 ng/mL in 79.9% of the sample. Additionally, 56.8% of the sample had CA125 values exceeding 30.2 U/mL, and 93.0% had APF values at or below 8.1 ng/mL.

Hormonal profiles were classified into low, normal, and high ranges. The analysis revealed that 77.8% of the patients had fT4 levels within the normal range (11.50–22.70 pmol/L), while 16.2% had fT4 levels greater than 22.70 pmol/L. For TSH, 72.3% of the patients had values within the normal range (0.55–4.78 mlU/L), and 18.1% had elevated TSH levels, exceeding 4.78 mlU/L. PTH levels were found to be within the normal range (18.5–88 pg/mL) in 43.8% of the patients, whereas half of these patients presented with elevated PTH levels (above 88 pg/mL).

Table 3 describes the association between CA 15-3 levels (classified as negative or positive) and the corresponding levels of CA125, CEA, AFP, fT4, and TSH among cases diagnosed with breast cancer in the observed cohort.

To investigate the association between the different biomarkers and blood parameters, classified according to reference ranges, either the chi-square test or Fisher’s exact test was employed. Specifically, Fisher’s exact test was used in instances where the expected frequencies were less than five.

The analysis revealed no statistically significant associations between CA 15-3 categories and the levels of CA125 (Pearson’s Chi-square, χ^2^(1) = 1.852, *p* = 0.174), CEA (Pearson’s Chi-square, χ^2^(1) = 1.139, *p* = 0.286), AFP (Fisher’s exact test, *p* = 0.341), fT4 (Fisher’s exact test, *p* = 0.310), TSH (Fisher’s exact test, *p* = 0.177), or PTH (Fisher’s exact test, *p* = 0.650). These findings indicate a lack of strong correlation between CA 15-3 and the various biomarkers among the cases studied.

**Figure 1:**
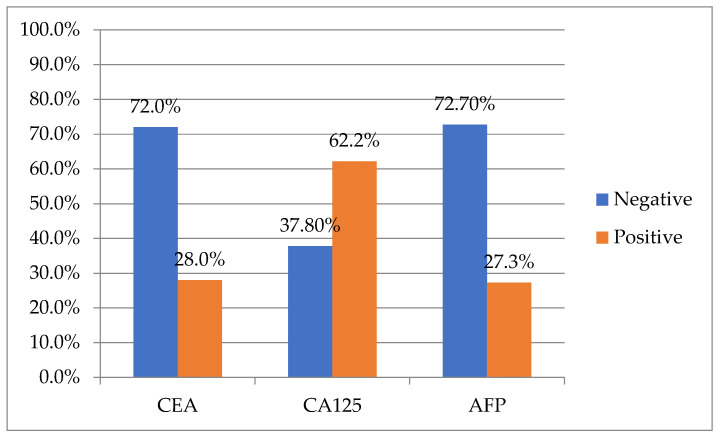
Distribution of patients with negative CA 15-3 in relation to CEA, CA125, and AFP levels.

**Figure 2:**
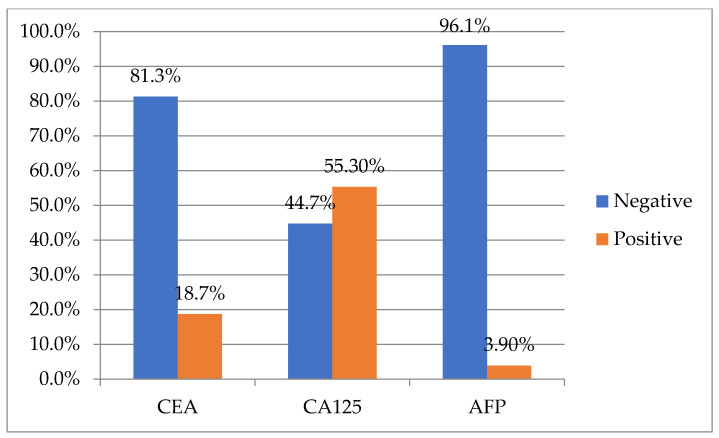
Distribution of patients with positive CA 15-3 in relation to CEA, CA125, and AFP levels.

Figure 1 and Figure 2 depict the distribution of patients with negative and positive CA 15-3 in relation to CEA, CA125, and AFP.

## 4. Discussion 

The mortality rate of breast cancer has decreased due to new diagnostic and therapeutic options. Clinicians may encounter therapeutic challenges regarding breast cancer management where prognostic and predictive biomarkers are valuable. The serum biomarkers, particularly CA 15-3 and CEA, can be very helpful in monitoring treatment response and early detection of recurrence or metastasis in breast cancer before imaging techniques show a tumor progression [45,46]. These serum biomarkers are useful for the follow-up of breast cancer patients due to their easy clinical availability, minimal invasiveness, dynamic tracking capabilities, and low cost [46,47]. 

Serum biomarker values are influenced by various non-tumorous factors: thereby, in some cases, increased levels of serum biomarkers may represent false positive results, leading to patient anxiety and unnecessary consumption of medical resources. Therefore, it is important for medical practitioners managing breast cancer patients to use serum biomarkers in the right clinical context [46].

CEA can also be raised in various nonmalignant conditions, such as benign hepatic and gastrointestinal diseases, chronic renal failure, and respiratory diseases [48]. Because it is predominantly metabolized in the liver, hepatic and biliary dysfunctions can be associated with elevated levels, causing false positives [49]. Some studies also report an association between raised CEA levels and metabolic syndrome [50,51]. CEA levels are typically elevated in smokers compared to nonsmokers [52].

CA15-3 can also be increased in nonmalignant liver diseases, chronic renal failure, colitis, and dermatological conditions [48]. Some other benign conditions that have been reported to raise CA15-3 levels are sarcoidosis, tuberculosis, and systemic lupus erythematosus [53]. Interstitial lung disease may also increase CA15-3 levels, a factor that should be considered when managing breast cancer patients treated with drugs known to induce this condition [54].

CA125 levels may also be raised in various benign conditions, such as acute or chronic liver diseases, pancreatitis, gastrointestinal diseases, respiratory diseases, acute urinary retention and chronic renal failure, heart failure, pericarditis, cystic fibrosis, diabetes, diseases of the female reproductive system, menstruation, pregnancy, peritoneal inflammation, sarcoidosis, systemic lupus erythematosus, arthritis, and recurrent ischemic strokes in patients with metastatic cancer [48]. The primary use of CA125 is in detecting and managing ovarian cancer [55].

AFP may be raised in liver regeneration and pregnancy [48]. Liver disease may also be a possible nonmalignant cause of AFP elevation [37]. It has been reported that elevated AFP does not correlate with breast cancer [56], while other studies suggest the contrary [57,58,59]. It has been reported that increased AFP levels are associated with a lower incidence of ER-positive breast cancer [60] and inhibit ER-positive cancer growth [61,62]. CEA and AFP could distinguish precancerous breast tissue from healthy controls, but further studies are needed to clarify this issue [59]. 

According to the literature, the available serum biomarkers lack sensitivity and specificity for early diagnosis, so their applicability in early-stage breast cancer screening or diagnosis is limited. The most known serum markers are MUC-1 family proteins (CA15-3, BR 27.29, MCA, and CA549), carcinoembryonic antigen (CEA), oncoproteins, and cytokeratins. The combination of one MUC-1 marker and CEA is used in breast cancer patients [62].

On the other side, there were many other tumor markers identified, such as DNA ploidy of the primary tumor as an independent prognostic factor for operable breast cancer, circulating plasma DNA levels in breast cancer patients, genetic markers BRCA1 and BRCA2 for identifying individuals who are at risk of developing breast and ovarian cancers, and tissue markers [62]. 

The supraphysiological expression of secreted proteins or their receptors represents cancer autocrine and paracrine signals. During malignant transformation, new signaling pathways are established through the disruption of the paracrine signaling networks [63].

Our work follows the possible correlations of serum tumor markers in breast cancer in relation to other blood parameters or glandular laboratory parameters. The available data regarding this issue is limited and conflicting. 

The studied tumor markers, namely, CA15-3, CEA, CA125, and AFP, have different physiological origins, and each becomes a marker for cancer due to its overexpression or aberrant presence in the context of tumor growth. As described above, these tumor markers have different patterns of expression. Our purpose was to see how other biomarkers, CEA, CA125, and AFP, and blood parameters of parathyroid and thyroid glandular dysfunction interplay with CA15-3, the most commonly used tumor marker in breast cancer.

According to our study, there were no significant associations found between serum biomarkers and other blood parameters; this is also sustained by the literature, which is often characterized by a lack of sensitivity and specificity. 

Not only CA 15-3 in breast cancer but also almost all serum markers lack sensitivity for low-stage invasive disease and specificity for detecting early breast cancer. However, no other markers have been shown to be superior to CA 15-3 in breast cancer, which is used to monitor response to treatment and detect recurrence in patients diagnosed with breast cancer [64,65]. CA 15-3 should be measured in conjunction with diagnostic imaging, clinical history, and physical examination, while serial determination can provide median lead times of 5–6 months [64]. CA 15-3 is one of the most important and reliable markers for metastatic breast cancer monitoring, usually detected in serum, but it can also be detected in saliva and cerebrospinal fluid [65,66]. CA 15-3 and CEA are the most widely used markers in breast cancer, although they are not recommended as screening markers [67]. Some studies show that serial determination of CA 15-3 and CEA levels in breast cancer patients may be useful for early detection of preclinical recurrence or metastatic disease [68,69]. Breast cancer is a heterogeneous group of diseases that require biomarkers for early detection of recurrence and metastatic disease [67]. According to some studies, elevation of CA 15-3 and CEA levels was associated with breast cancer of molecular subtype, being often increased in the luminal subtypes of breast cancer and correlated with bone metastasis. The elevation in CA 15-3 levels varies according to the number of metastatic sites, whereas elevated CEA was observed regardless of the metastatic site [67]. In our study, we found no strong association between CA 15-3 and CEA values, which may suggest that these markers vary independently in the context of breast cancer. CA 15-3 levels can rise in various types of cancer, including those of the breast, ovary, lung, liver, or pancreas. However, elevated CA 15-3 can also occur in benign conditions affecting the liver, breast, and ovary [70]. Similarly, CEA levels can increase in cancers such as colorectal, breast, liver, and pancreatic cancer, as well as in some non-cancerous conditions, for example, in smokers. Notably, in these non-malignant scenarios, an increase in CEA above 10 ng/mL is uncommon [71]. The levels of CA 15-3 and CEA can be influenced by several factors, including the type and stage of cancer, the presence of metastases, and individual patient factors.

According to the literature, tumor marker combinations are more reliable than single detections and can be used as auxiliary tools in the diagnosis of breast cancer [7]. The current primary application for serum markers in breast cancer is to aid in evaluating the response to chemotherapy in advanced-stage disease [72].

Our findings and the few existing data points in the literature indicate that there may be no correlation between CA 15-3 and parathyroid hormone [39]. It has been reported that women with a history of primary hyperparathyroidism have a similar breast cancer prognosis compared to women without primary hyperparathyroidism [73].

The relationship between breast cancer and thyroid cancer is not fully understood. Autoimmune thyroiditis with subclinical or manifest hypothyroidism is frequently found in women with breast cancer. The literature shows that increased serum levels of thyroid-stimulating hormone (TSH) with subclinical or manifest hypothyroidism were found in 10.0–19.7% of women with breast cancer [74]. The thyroid axis in breast cancer may represent a therapy target, involving the presence of thyroid hormone receptors in breast tumors as well as the interaction between thyroid hormones and ER [40,75].

On the other side, in terms of personalized therapy and optimizing clinical practice, the literature shows that mammographic and sonographic imaging and magnetic resonance imaging (MRI) are not only important tools for testing the chemotherapy tumor response but can also be associated with breast cancer molecular subtypes [76,77,78]. Maybe future studies could consider correlating serum tumor markers with other predictive factors in breast cancer, such as imaging data or the ABO blood group. Although the ABO blood group is a risk factor for several cancers [79], some studies showed that there was no correlation between ABO blood type genotypes/phenotypes and breast cancer [79].

Circulating tumor markers have limitations in sensitivity and specificity, especially in the early stages of malignant disease. Diagnostic accuracy and risk stratification can be achieved by combining multiple markers, while challenges in clinical implementation include standardization, validation, and algorithm development [80].

Limitations of the study include the susceptibility to confounding variables and an imbalanced sample size between categories, which may impact the interpretation and generalizability of our findings. To address these limitations and enhance the robustness of the analysis, future studies could consider larger sample sizes with balanced representation across categories and include additional relevant variables.

## 5. Conclusions 

In summary, the analysis conducted on the levels of CA125, CEA, AFP, fT4, or TSH compared to CA 15-3 levels in cases diagnosed with breast cancer within this specific cohort did not identify any statistically significant associations. Based on our findings, we hypothesize that CA 15-3 levels do not exhibit a strong relationship or dependence on the levels of CA125, CEA, AFP, fT4, or TSH. In order to validate this conclusion, further investigations in larger cohorts or prospective studies with serial measurements of markers are recommended.

## Figures and Tables

**Table 1:** Mean, median, standard deviation, and minimum and maximum values for each variable measured.

	CA 15-3	CEA	CA125	AFP	fT3	fT4	TSH	Cortisol	PTH
N	231	164	146	114	32	99	83	9	16
Mean	111.42	24.18	337.63	361.79	4.15	18.07	3.35	29.51	163.47
SD	317.74	100.11	916.43	2918.29	1.32	5.50	3.44	17.55	175.34
Minimum	4.50	0	0	0	1.34	10.10	0.10	10.40	7.90
Maximum	4000.00	1130.0	5530.00	29,800.00	7.04	41.86	14.10	70.66	672.50

N = number of cases; SD = standard deviation.

**Table 2:** Frequencies for CA 15-3, CEA, CA125, AFP, fT4, TSH, and PTH categories.

	Negative		Positive	
CA 15-3 (N = 231)	38 (16.5%)		193 (83.5%)	
CEA (N = 164)	131 (79.9%)		33 (20.1%)	
CA125 (N = 146)	63 (43.2%)		83 (56.8%)	
AFP (N = 114)	106 (93.0%)		8 (7.0%)	
	Low	Normal		High
fT4 (N = 99)	6 (6.1%)	77 (77.8%)		16 (16.2%)
TSH (N = 83)	8 (9.6%)	60 (72.3%)		15 (18.1%)
PTH (N = 16)	1 (6.3%)	7 (43.8%)		8 (50.0%)

**Table 3:** Association between studied CA 15-3 and CA125, CEA, AFP, fT4, TSH, and PTH.

Variables		Negative CA 15-3	Positive CA 15-3	Test Statistic, *p*-Value
CEA	Negative CEA	18 (72.0%)	113 (81.3%)	Pearson’s Chi-square, *χ*^2^(1) = 1.139, *p* = 0.286
	Positive CEA	7 (28.0%)	26 (18.7%)	
CA125	Negative CA125	4 (37.8%)	59 (44.7%)	Pearson’s Chi-square, *χ*^2^(1) = 1.852, *p* = 0.174
	Positive CA125	11 (62.2%)	72 (55.3%)	
AFP	Negative AFP	15 (72.7%)	91 (96.1%)	Fisher’s exact test, *p* = 0.341
	Positive AFP	2 (27.3%)	6 (3.9%)	
fT4	Low fT4	2 (15.4%)	4 (4.7%)	Fisher’s exact test, *p* = 0.310
	Normal fT4	9 (69.2%)	68 (79.1%)	
	High fT4	2 (15.4%)	14 (16.3%)	
TSH	Low TSH	3 (25.0%)	5 (7.0%)	Fisher’s exact test, *p* = 0.177
	Normal TSH	7 (58.3%)	53 (74.6%)	
	High TSH	2 (16.7%)	13 (18.3%)	
PTH	Low PTH	0 (0.0%)	1 (7.7%)	Fisher’s exact test, *p* = 0.650
	Normal PTH	2 (6.7%)	5 (38.5%)	
	High PTH	1 (3.3%)	7 (53.8%)	

## Data Availability

Further information concerning the present study is available from the corresponding author upon reasonable request.
